# *Ex vivo* imaging of subacute myocardial infarction with ultra-short echo time 3D quantitative T_1_- and T_1*ρ*_-mapping magnetic resonance imaging in mice

**DOI:** 10.1093/ehjimp/qyae131

**Published:** 2024-12-30

**Authors:** Iida Räty, Antti Aarnio, Mikko J Nissi, Sanna Kettunen, Anna-Kaisa Ruotsalainen, Svetlana Laidinen, Seppo Ylä-Herttuala, Elias Ylä-Herttuala

**Affiliations:** A.I. Virtanen Institute, University of Eastern Finland, Neulaniementie 2, 70210 Kuopio, Finland; Department of Technical Physics, University of Eastern Finland, Yliopistonranta 8, 70210 Kuopio, Finland; Department of Technical Physics, University of Eastern Finland, Yliopistonranta 8, 70210 Kuopio, Finland; A.I. Virtanen Institute, University of Eastern Finland, Neulaniementie 2, 70210 Kuopio, Finland; A.I. Virtanen Institute, University of Eastern Finland, Neulaniementie 2, 70210 Kuopio, Finland; A.I. Virtanen Institute, University of Eastern Finland, Neulaniementie 2, 70210 Kuopio, Finland; A.I. Virtanen Institute, University of Eastern Finland, Neulaniementie 2, 70210 Kuopio, Finland; Heart Center, Kuopio University Hospital, Puijonlaaksontie 2, 70210 Kuopio, Finland; A.I. Virtanen Institute, University of Eastern Finland, Neulaniementie 2, 70210 Kuopio, Finland; Clinical Imaging Center, Kuopio University Hospital, Puijonlaaksontie 2, 70210 Kuopio, Finland

**Keywords:** cardiac magnetic resonance, quantitative imaging, myocardial infarction, compressed sensing, 3D imaging, ultra-short echo time

## Abstract

**Aims:**

The aim of this study was to develop an ultra-short echo time 3D magnetic resonance imaging (MRI) method for imaging subacute myocardial infarction (MI) quantitatively and in an accelerated way. Here, we present novel 3D T_1_- and T_1*ρ*_-weighted Multi-Band SWeep Imaging with Fourier Transform and Compressed Sensing (MB-SWIFT-CS) imaging of subacute MI in mice hearts *ex vivo*.

**Methods and results:**

Relaxation time–weighted and under-sampled 3D MB-SWIFT-CS MRI were tested with manganese chloride (MnCl_2_) phantom and mice MI model. MI was induced in C57BL mice, and the hearts were collected 7 days after MI and then fixated. The hearts were imaged with T_1_ and adiabatic T_1*ρ*_ relaxation time–weighted 3D MB-SWIFT-CS MRI, and the contrast-weighted image series were estimated with a locally low-rank regularized subspace constrained reconstruction. The quantitative parameter maps, T_1_ and T_1*ρ*_, were then obtained by performing non-linear least squares signal fitting on the image estimates. For comparison, the hearts were also imaged using 2D fast spin echo-based T_2_ and T_1*ρ*_ mapping methods. The relaxation rates varied linearly with the MnCl_2_ concentration, and the T_1_ and T_1*ρ*_ relaxation time values were elevated in the damaged areas. The ischaemic areas could be observed visually in the 3D T_1_, 3D T_1*ρ*_, and 2D MRI maps. The scar tissue formation in the anterior wall of the left ventricle and inflammation in the septum were confirmed by histology, which is in line with the results of MRI.

**Conclusion:**

MI with early fibrosis, increased inflammatory activity, and interstitial oedema were determined simultaneously with T_1_ and T_1*ρ*_ relaxation time constants within the myocardium by using the 3D MB-SWIFT-CS method, allowing quantitative isotropic 3D assessment of the entire myocardium.

## Graphical Abstract

**Graphical abstract:**
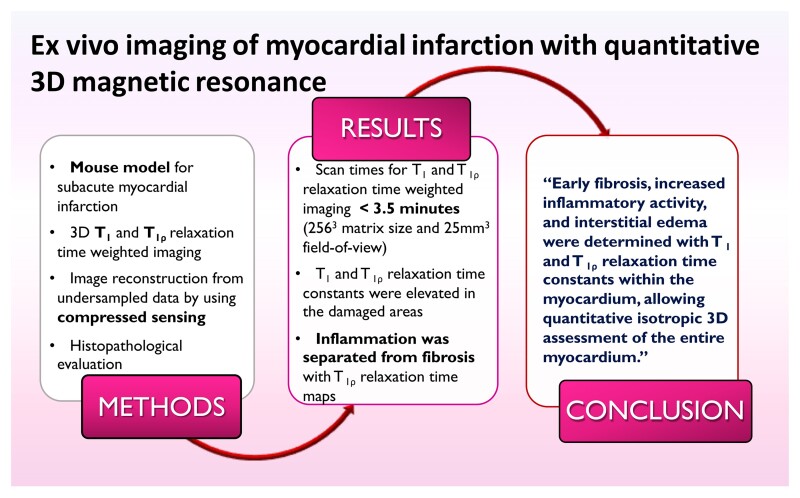

## Introduction

Myocardial infarction (MI) is one of the most severe consequences of coronary artery disease, caused by the occlusion of coronary arteries. Ischaemia in the myocardium activates cell stress signals and an inflammatory response, leading to the infiltration of inflammatory cells to activate tissue repair processes, which stimulates collagen synthesis and eventually causes fibrotic scar formation in the infarcted area.^1,2^

Magnetic resonance imaging (MRI) has a vital role in the diagnosis and follow-up of cardiovascular diseases (CVDs) and their complications.^1,3,4^ The advantages of MRI over current radiation- and nuclear-based cardiac imaging techniques include high soft tissue contrast and high spatial resolution. Gadolinium-based contrast agents (GBCAs) are widely used in cardiac magnetic resonance (CMR) for determination of MI area.^5^ GBCAs accumulate unspecifically in the extracellular space^5,6^ resulting only in the visualization of extracellular regions. As an alternative, multiple methods for mapping endogenous contrast mechanisms, i.e. relaxation times, have been developed.^5,7,8^ Endogenous relaxation 2D MRI mapping methods, e.g. T_1_, T_2_, and T_1*ρ*_, are used for the assessment of CVDs by revealing damaged tissue from remote and healthy tissue.^8–12^ Moreover, quantitative 3D CMR methods have also been developed.^13–15^ T_1*ρ*_ has been shown to be sensitive to macromolecular interactions, indicating that T_1*ρ*_ could be a quantitative MRI marker for tissue remodelling after MI, where oedema, granulation tissue, and replacement fibrotic tissue have developed.^10,16^ In clinics, CMR is mainly performed as 2D multi-slice imaging with reduced spatial coverage. Therefore, 3D imaging with high isotropic resolution would be highly desirable. Multi-Band SWeep Imaging with Fourier Transform (MB-SWIFT)^17^ is a radial 3D MRI sequence, in which the echo time (TE) can be made extremely short (≈0) together with large excitation and acquisition bandwidths. The centre-out radial acquisition scheme makes MB-SWIFT resistant to motion artefacts, which is essential in imaging of the heart.^17–19^ MB-SWIFT can also be utilized as a readout sequence for different contrasts by implementing contrast manipulations using magnetization preparation (MP) blocks in the sequence.^20–22^ The extremely short TE of MB-SWIFT allows capturing even the very fast T_2_ relaxation,^17^ which is used to characterize oedema in acute MI.^9,23^ Therefore, MB-SWIFT could be useful when an acute MI is defined. Surprisingly, MB-SWIFT is still somewhat unused in the field of CMR since the earlier 3D SWIFT, a variant of which MB-SWIFT is, has only been applied to track labelled stem cells in rat hearts.^24^

Furthermore, MRI tends to be slow, which limits its use. The motion of the heart during the scanning usually means that either non-optimal image quality or long imaging times must be tolerated in traditional and especially in 3D CMR. One way to accelerate MRI is to utilize compressed sensing (CS).^25,26^ CS leverages the sparsity of the true image and under-sampling trajectories that produce noise-like artefacts to estimate a good-quality image from an incomplete set of measurements. CS has been used in various CMR studies^25–28^ and with the MB-SWIFT sequence.^29^

In this study, we employed a manganese chloride (MnCl₂) phantom to test our 3D T_1_- and T_1*ρ*_-weighted MB-SWIFT-CS method. The same methodology was applied in mice *ex vivo* hearts to further assess the imaging technique’s applicability in a more complex biological system. Finally, the mice hearts were scanned 7 days after MI with 3D T_1_, 3D T_1*ρ*_, and 2D relaxation time methods. Relaxation methods were used to assess the potential for differentiating between pathologies in the myocardium after MI.

## Methods

### Phantom

The phantom study allows the assessment of the accuracy and robustness of the relaxation time–weighted MB-SWIFT-CS method in quantifying relaxation time constants under known conditions. The phantom was constructed using four 0.25 mL eppendorf tubes. A 1-mM MnCl_2_ solution was prepared and diluted to obtain the following concentrations: 0.01, 0.03, 0.05, and 0.09 mM. The tubes were labelled, filled with mixtures of varying concentrations, and imaged with quantitative 3D and 2D MRI methods.

### Animal model

The purpose of using the animal model is to compare the T_1_ and T_1*ρ*_ relaxation time constants between infarcted, remote, and healthy myocardium. This comparison helps identify changes in tissue properties caused by MI and evaluate the potential of the relaxation time–weighted MB-SWIFT-CS method for detecting myocardial damage.

C57BL mice (*n* = 8) were housed in the Animal Centre of the University of Eastern Finland, where they received food and water *ad libitum*. To induce MI, the left anterior descending (LAD) coronary artery was surgically ligated with the so-called pop-up technique. In the procedure, the heart was removed extracorporeally, ligated, and then reimplanted.^30^ During the operation, mice were under isoflurane inhalation anaesthesia (4% induction, 2% maintenance) and received analgesia during the operation and for the following 2 days (5 mg/kg carprofen once a day and 0.1 mg/kg buprenorphine twice a day). After 7 days, mice were sacrificed with CO_2_, and the hearts were collected and fixed in 4% paraformaldehyde (PFA) in phosphate buffered saline overnight. During the imaging, the hearts were kept in perfluoropolyether (Galden HS 240, Solvay Solexis, Bollate, Italy).

**Figure 1:**
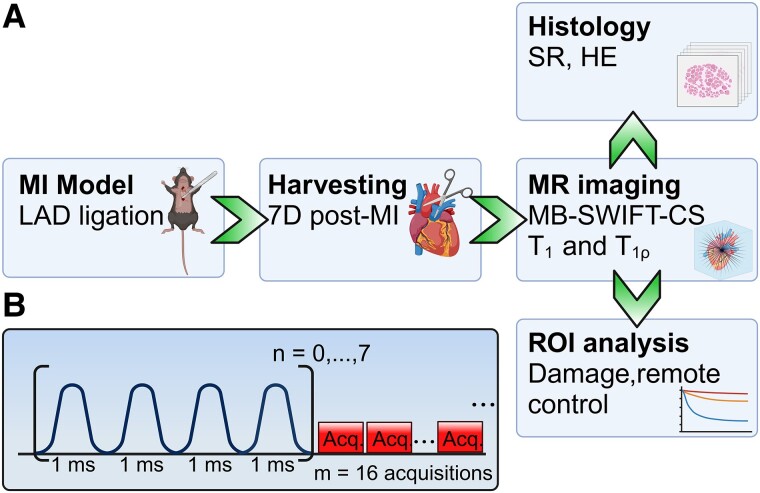
(*A*) Study framework. MI mouse model was used, and the hearts were harvested 7 days after the operation and imaged with T_1_- and T_1*ρ*_-weighted MB-SWIFT sequence. Image reconstruction was performed by utilizing CS. After the imaging, ROI analysis was performed to damaged and remote myocardium by utilizing relaxation time maps. Histology was performed by using SR and HE stainings. (*B*) T_1*ρ*_ sequence design. The magnetization block consisted of 4 AFP, and blocks were repeated every 16 spokes during the imaging sequence. Acq, acquisition. Created in BioRender, Räty, I. (2025) https://BioRender.com/u65o579

Additionally, hearts from three non-operated mice were collected for the control group. The framework of the animal study is presented in *Figure 1*.

### MRI

The CMR was performed with a vertical pre-clinical 9.4 T/89 mm magnet connected to a console (VnmrJ DirectDrive software v.3.2, Varian Associates Inc., Palo Alto, CA, USA) and a 15-mm quadrature radio frequency volume transceiver coil (Rapid, Biomedical GmbH, Rimpar, Germany). In 3D MRI measurements, total acquisition times were, and corresponding acquisition times were calculated based on the amount of data used in the image reconstruction.

#### 3D T_1_ measurements

For T_1_-weighted imaging, a 3D MB-SWIFT sequence was employed, utilizing an adiabatic half passage (AHP) pulse with tanh/tan modulation [duration = 500 µs, radiofrequency (RF) power *ω*_1,max_/2*π* = 4100 Hz] as a saturation pulse after every 1024 spokes for T_1_ measurement as described previously,^21^ with the following MB-SWIFT sequence parameters: acquisition time for a single spoke *τ* = 3 ms, flip angle (FA) 2° (for phantom), field of view (FOV) 50 mm^3^, matrix size 256^3^, total number of spokes = 1 048 576, and spokes between saturation pulses, i.e. per single look-locker curve = 1024.^31–34^ For the hearts, FA was increased to 4° to increase the signal-to-noise ratio (SNR) and the FOV was set to 25 mm^3^. For the image reconstruction, the data were retrospectively under-sampled, and only the first 65 536 spokes were used. The resulting 64 look-locker curves were binned into 32 images. Each of the images, thus, comprised of 2048 spokes, and the image series was then reconstructed with CS. The acquisition corresponds to a scan time of 3.25 min for the whole heart.

#### 3D T_1*ρ*_ measurements

For determination of T_1*ρ*_, a train of frequency-modulated adiabatic full-passage (AFP) preparation pulses was used for the MP.^20^ MB-SWIFT sequence with FA = 3° was used as a readout sequence. Eight 3D MB-SWIFT images were acquired with the same gain settings, but different spin-lock durations (TSLs) (T_mp_) were set at 0, 4, 8, 12, 16, 20, 24, and 28 ms. The preparation involved the use of MP blocks, each incorporating an increasing number (*n* = 0–7) of 4-step Malcolm Levitt phase cycling-4 (MLEV-4) phase-cycled sets. Each set consisted of four AFP pulses of the hyperbolic secant (Hypersecant pulse family n) family with a stretching factor of *n* = 1 and with a time-bandwidth product value of *R* = 10 (duration of each pulse = 1000 µs, RF power *ω*_1,max_/2*π* = 3700 Hz).^35^ These MP blocks were repeated every 16 spokes during the imaging sequence. Although 65 536 spokes were collected per set, only 6553 spokes per image were utilized in the CS image reconstruction, corresponding to a total 3D scan time of 3.44 min. The illustration of the T_1*ρ*_ sequence design is shown in *Figure 1*.

#### 2D MRI

After the MB-SWIFT imaging, the phantom and the hearts were scanned immediately with a 2D quantitative MRI. Imaging was done in one 1-mm thick axial slice at the lower mid-level of the heart and approximately through the centres of the phantom tubes with fast spin echo (FSE) coupled to adiabatic T_1*ρ*_ and T_2_, contrasts as performed in the study by Yla-Herttuala et al.^8^ For 2D adiabatic T_1*ρ*_ imaging, an FSE sequence was utilized with increasing trains of four HS4 AFP pulses with *R* = 20 as contrast preparation, similar to the 3D acquisition, with each pulse in the train lasting 2 ms at RF power *ω*_1,max_/2*π* = 2500 Hz. The readout sequence had a repetition time (TR) of 5 s, TE of 10 ms, and matrix size of 192². The FOVs were set to 32 and 20 mm² for the phantom and the hearts, respectively. By increasing the number of pulses in the train, the TSLs were set to 0, 9.1, 18.1, and 36.2 ms. For T_2_-weighted imaging, the same 2D FSE readout sequence was used with an initial AHP pulse of 4 ms at 2500 Hz, followed by two HS1 *R* = 10 pulses of 4 ms each at 2500 Hz and concluded with a reversed AHP pulse, with the pulses separated by TE/4, TE/2, and TE/4. The TEs for the contrast preparation part of this sequence were set to 0.05, 2.3, 4.5, and 14 ms.

### Image reconstruction

The T_1_-weighted image series was reconstructed by utilizing a locally low-rank (LLR)^36^ regularized subspace constrained image reconstruction^37^ (Appendix 1 Eq. A.1). The T_1*ρ*_ images were reconstructed with the LLR regularization but without subspace projection (Appendix 1 Eq. A.3). The LLR regularization block size was 8^3^. The appropriate regularization strength was chosen based on visual inspection of the T_1_ or T_1*ρ*_ relaxation time maps with particular attention to preserving image details. All reconstruction models were solved with 70 iterations of the pre-conditioned primal-dual proximal splitting algorithm.^38,39^ The algorithm was implemented in Python,^40–42^ and reconstruction times were noted.

### Data analysis

Relaxation time map calculations and regions of interest (ROIs) analyses were performed with MATLAB R2022b (MathWorks Inc., Natick, CA, USA), using Aedes software (http://aedes.uef.fi).

#### Relaxation time mapping using 3D MB-SWIFT

Effective T_1_ (T_1eff_) maps were generated pixel-by-pixel by utilizing a five-parameter non-linear least squares fitting.^21^ The T_1_ relaxation time constants were then calculated from T_1eff_ values by using a FA-based correction Equation B2 (Appendix 2).^21,32^

The estimated T_1_ relaxation time maps were also utilized in the calculation of T_1*ρ*_ relaxation time maps by following the derivation presented in the study by Zhang *et al*.^20^ After normalization to correct for the T_1_ effects, the same signal equation B3 presented in^20^ was used. From the normalized signal equation (B4), T_1*ρ*_ relaxation time constant was solved by performing mono exponential non-linear least squares fitting (see Appendix 2 for equations).

Besides the single-component approach, T_1*ρ*_ relaxation time constant was also studied in a multi-component manner by calculating so-called asymptotic average relaxation time T_1*ρ*a_ and slow relaxation time T_1*ρ*s_ values as presented in.^20^ The T_1*ρ*a_ was determined with a slope, which was achieved by fitting the line over the first two data points after the data were normalized and presented in a logarithmic scale. T_1*ρ*s_ values were determined similarly, but only the remaining data points were used.

#### 2D relaxation time mapping

Signal intensities were fit using the standard mono exponential signal model, using the same least squares approach as used in the study by Yla-Herttuala *et al*.^8^

### Relaxation time analysis

Relaxation time analysis was performed by using 3D T_1_ and 3D T_1*ρ*_ relaxation time maps. For the MnCl_2_ phantom, T_1_ and T_1*ρ*_ relaxation time maps were collected, and relaxivity was determined as a slope of a linear fit of relaxation rate *R* (=1/T_1_ or 1/T_1*ρ*_) and concentration. For the hearts, the co-registration between 2D image slices and 3D MB-SWIFT-CS short-axis (SAX) view images was performed based on the anatomical structures of the heart, i.e. the shape and size of the papillary muscles and left ventricle (LV). To avoid biases, the ROIs were drawn to the anatomical images and then saved as a template. Subsequently, the ROI template was used to collect the calculated average T_1_ and T_1*ρ*_ relaxation time constants. ROIs were drawn to the damaged and remote areas, which were visually observed from the 2D T_2_ and T_1*ρ*_ maps. Additionally, relative relaxation time difference (RRTD) was calculated for all relaxation time maps as follows:

Equation 1


$$\mathrm{RRTD}=\frac{\mathrm{mean}\;(T_{\alpha ,\mathrm{damage}})-\mathrm{mean}\;(T_{\alpha ,\mathrm{remote}})}{\mathrm{mean}\;(T_{\alpha ,\mathrm{remote}})},$$

Where T*_α_* is either T_1_, T_1*ρ*_, or T_2_.

### Histology

After the hearts were imaged, standard tissue processing and paraffin embedding methods were used. Four-micrometre thick cross-sections of the hearts were cut and stained with haematoxylin and eosin (HE) and PicroSirius Red (Abcam, Cambridge, UK) by following the manufacturer’s protocols.^43^ The slides were mounted with Permount (ThermoFisher Scientific). Sections were photographed using a Nikon Eclipse microscope with a DsRi2 camera (Nikon Instruments Europe BV). Tissue oedema was analysed by digitally quantifying the empty extracellular space in the myocardium by using the colour threshold method of the Fiji image processing software.^44^ The average percentage of the septum oedema area of the MI hearts was then compared with the septum of healthy hearts.

### Statistical analysis

The structural changes between remote and damaged areas were compared using a *t*-test with Bonferroni correction. The groups consisted of mean values from ROIs, and normality was assessed using the Shapiro–Wilk test for both the damage and remote groups. The control group was excluded from the analysis due to the limited sample size. Statistical analysis was performed using MATLAB and GraphPad Prism v5.03 (GraphPad Software Inc.), with a *P*-value of <0.05 considered statistically significant.

## Results

### Phantom measurements

**Figure 2:**
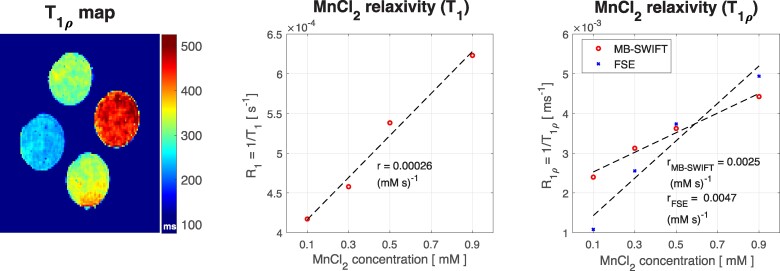
T_1*ρ*_ map and relaxation rates as a function of the MnCl_2_ concentration. Relaxivities *r*_1,_  *r*_MB-SWIFT_, and *r*_FSE_ were determined with a linear fit.

T_1_ and T_1*ρ*_ relaxation time constants were determined for different MnCl_2_ concentrations from the phantom measurements. For MB-SWIFT, the average T_1_ relaxation time constants for 0.1, 0.3, 0.5, and 0.9 mM MnCl_2_ concentrations were 2.4, 2.2, 1.9, and 1.6 s, respectively. T_1*ρ*_ relaxation time constants were 420, 320, 280, and 230 ms for the same concentrations. For FSE, T_1_ relaxation time constants were 2.0, 1.8, 1.6, and 1.4 s, and T_1*ρ*_ relaxation time constants were 920, 390, 270, and 200 ms for concentrations from 0.1 to 0.9 mM. Additionally, relaxivity *r* of the MnCl_2_ was determined from T_1_ and T_1*ρ*_ MB-SWIFT measurements (*Figure 2*), resulting in *r*_1_ = 0.00024 (mM s)^−1^, *r*_MB-SWIFT_ = 0.0025 (mM s)^−1^, and *r*_FSE_ = 0.0047 (mM s)^−1^ in 9.4 T.

### Image reconstruction

**Figure 3:**
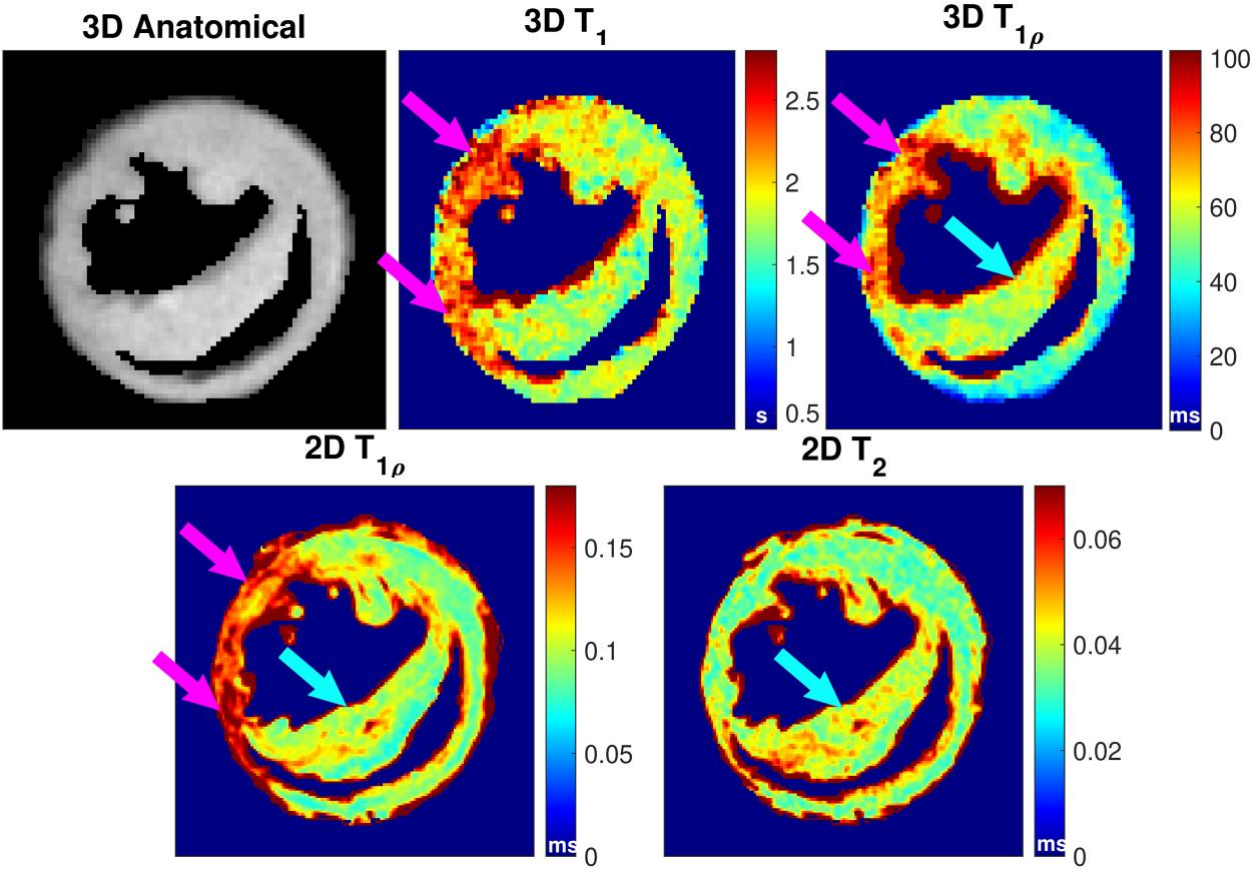
View from the 3D anatomical image and corresponding T_1_ and T_1*ρ*_ maps 7 days after MI. For a comparison, one slice was acquired with conventional 2D T_1*ρ*_ and 2D T_2_ methods and utilized in the ROI analysis. Pink arrows are pointing to the left ventricular wall and blue arrows are pointing to the septum. Arrows pointing to the left ventricular wall (pink) and the septum (blue) show the regions where fibrosis and inflammation were detected with histology, respectively.

**Figure 4:**
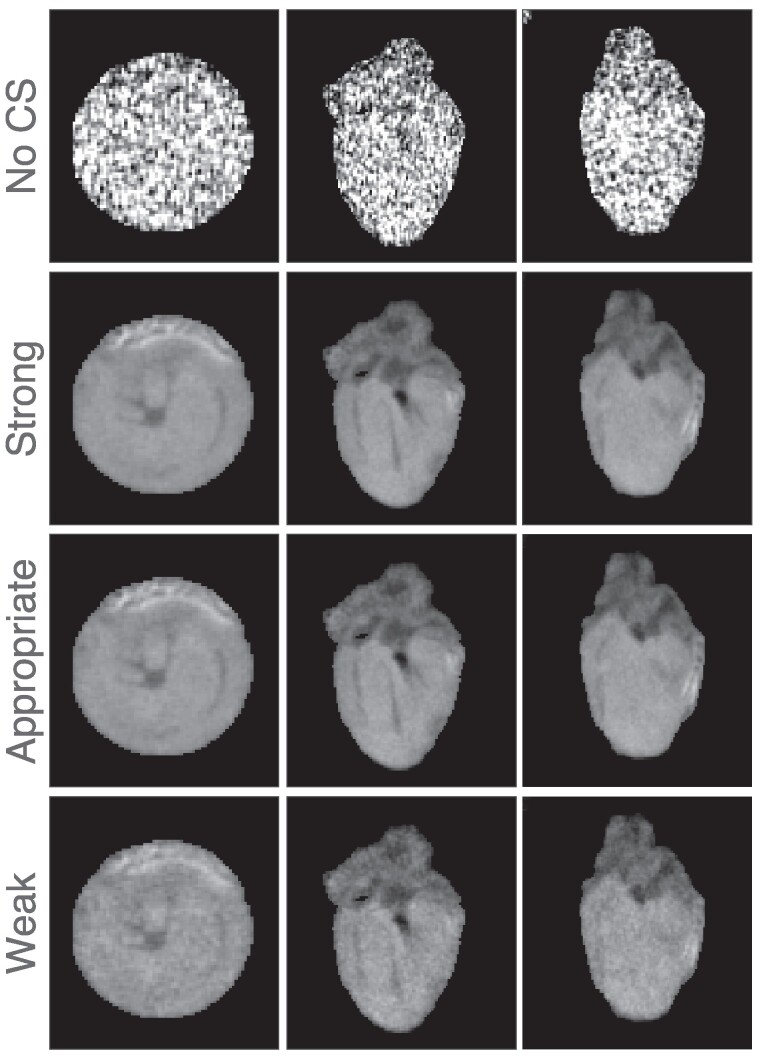
The impact of CS. The top row displays images obtained without LLR regularized subspace reconstruction. Below, images reconstructed using LLR with varying regularization strength (weak, appropriate, and strong) are presented.

**Figure 5:**
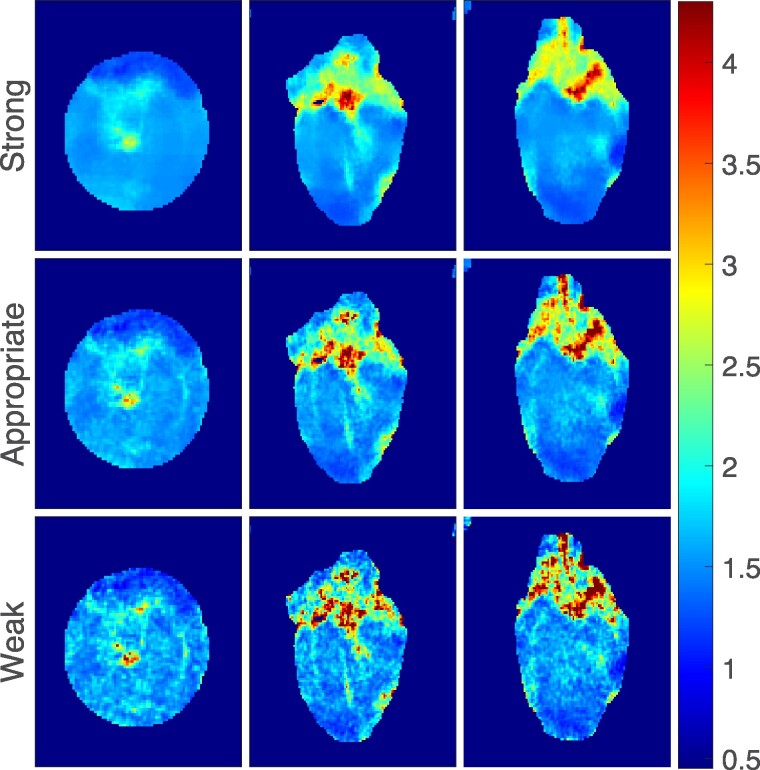
Calculated T_1_ maps derived from MB-SWIFT-CS measurements, using the image reconstructed with strong, appropriate, and too weak regularization strengths. Unit for colorbar is second.

The anatomical image and the calculated T_1_ and T_1*ρ*_ relaxation time maps from MB-SWIFT-CS measurements are shown in *Figure 3*. The comparison of the anatomical images with different regularization parameters and the resulting T_1_ maps are presented in *Figures 4* and *5*, respectively. Too strong regularization resulted in blurry images, and the effect was emphasized in the calculated relaxation time maps. On the other hand, too weak regularization resulted in noisy images (*Figure 5*). The image reconstruction with CS resulted in a corresponding scan time of 3.25 min for 3D T_1_ measurements and 3.44 min for 3D T_1*ρ*_ measurements. The image reconstruction was performed off-line, and it took ∼10–30 min on a dual Xeon E5-2630 v3 @ 2.4 GHz server depending on server load.

### Quantitative MRI revealed damage in the MI area

**Figure 6:**
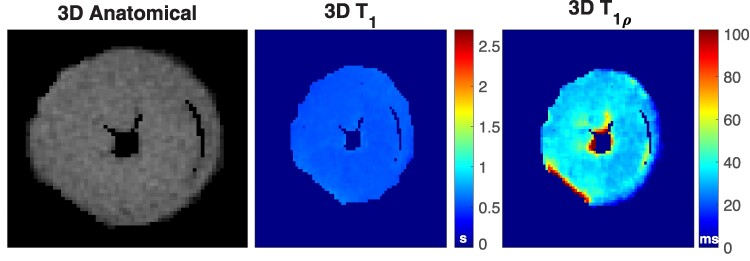
Healthy mouse heart imaged with MB-SWIFT-CS. Anatomical image (left), calculated T_1_ (middle), and T_1*ρ*_ (right) maps from MB-SWIFT-CS measurements.

In 3D T_1*ρ*_ measurements, some small details were better visualized in the single-component T_1*ρ*_ images rather than in T_1*ρ*a_ or T_1*ρ*s_ maps, and thus, T_1*ρ*_ data were used in the analysis. The 3D anatomical images, 3D T_1_ and 3D T_1*ρ*_, 2D T_1*ρ*_, and 2D T_2_ maps were used in the ROI analysis (*Figure 3*). The ROI analysis indicated that both 3D T_1_ and 3D T_1*ρ*_ relaxation time constants were significantly elevated in the LV wall compared with remote and control tissues (*Figures 3* and  *6*) For 3D T_1_ measurements, the mean values of ROIs from control, remote, and damaged areas were 0.68 ± 0.042, 1.4 ± 0.21, and 1.9 ± 0.31 s, respectively. For 3D and 2D T_1*ρ*_ measurements, mean values of ROIs from control, remote, inflammation, and fibrosis areas were 30 ± 2.6, 31 ± 3.2, 50 ± 5.7, and 84 ± 12 for 3D measurements and 63 ± 0.050, 74 ± 5.2, 110 ± 4.5, and 153 ± 18 for 2D T_1*ρ*_ measurements. For 2D T_2_ measurements, mean T_2_ relaxation time values from the control, remote, and oedema ROIs were 31 ± 0.050, 31 ± 0.82, and 61 ± 8.4. Values are also presented in *Table 1*.

**Table 1:** Collected relaxation time values from 3D T_1_, T_1***ρ***_, **and 2D T_1*ρ*_ and T_2_ measurements**

Contrast	Control (mean ± std)	Remote (mean ± std)	Inflammation/oedema (mean ± std)	Fibrosis (mean ± std)
3D T_1_ (s)	0.68 ± 0.042	1.4 ± 0.21	^a^	1.9 ± 0.31
3D T_1*ρ*_ (ms)	30 ± 2.6	31 ± 3.2	50 ± 5.7	84 ± 12
2D T_1*ρ*_ (ms)	63 ± 0.050	74 ± 5.2	110 ± 4.5	153 ± 18
2D T_2_ (ms)	31 ± 0.050	31 ± 0.82	61 ± 8.4	^a^

Average values of the region of interest are shown.

^a^Not investigated.

**Figure 7:**
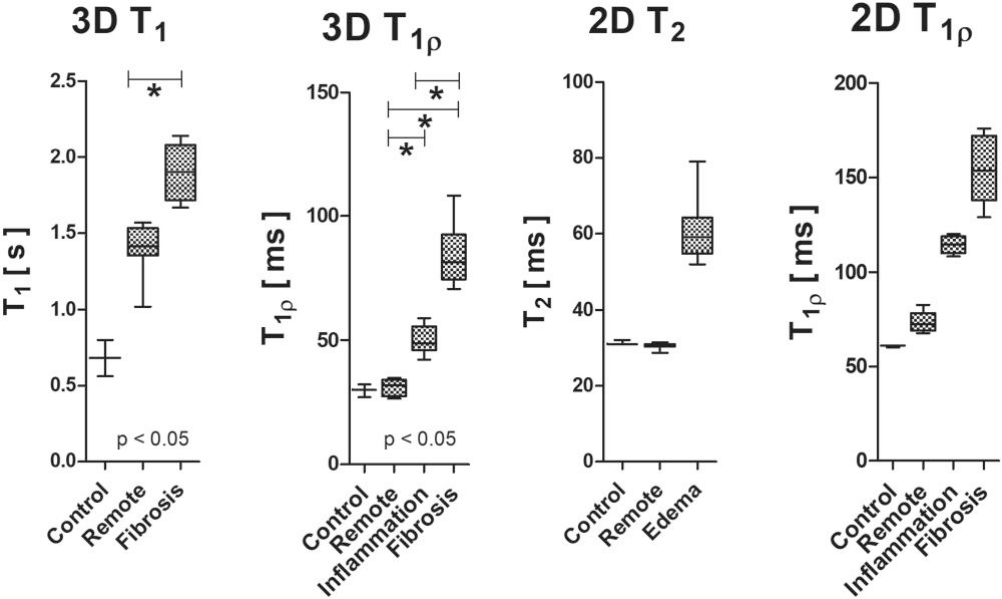
Boxplots showing the distribution of 3D T₁, 3D T_1*ρ*_, 2D T_1*ρ*_, and 2D T₂ relaxation times across different ROIs (control, remote, and damaged). Statistical analysis was performed for 3D data, and significant differences (*P* < 0.05) are marked with an asterisk. The data indicate distinct relaxation time profiles for different tissue types, with notable increases in relaxation times in damaged areas compared with control tissues.

The analysis of T_1_ relaxation time constants between remote and damaged ROIs revealed a statistically significant difference with a *t*-test (*P* < 0.00008). Additionally, the *t*-test indicated significant differences between the remote and inflammation ROIs (*P* < 0.000002), the remote and fibrosis ROIs (*P* < 0.000003), and the fibrosis and inflammation ROIs (*P* < 0.00006; *Figure 7*).

In the 3D T_1*ρ*_ imaging, RRTD of 1.7 was observed between fibrotic and remote tissues (*Table 2*). The contrast difference for inflammation vs. remote tissue was 0.63, and the contrast difference between fibrosis and inflammation was 0.67 with 3D T_1*ρ*_ (*Table 2*). The analysis revealed that 3D T_1_ imaging provided a contrast difference of 0.37 when distinguishing fibrotic tissue from remote tissue (*Table 2*). For 2D T_1*ρ*_ imaging, the contrast difference between fibrotic and remote tissues was 1.1, between inflamed and remote tissues was 0.55 and between fibrosis and inflammation was 0.35 (*Table 2*). In T_2_ measurements, the contrast differences between tissue with oedema and remote tissue and remote and control tissues were 0.99 and −0.025, respectively (*Table 2*).

**Table 2:** Calculated RRTD values

Comparison→	Fibrosis/remote	Inflammation/remote	Fibrosis/inflammation	Remote/control
contrast↓				
3D T_1_	0.37	^a^	^a^	0.68
3D T_1*ρ*_	1.7	0.63	0.67	0.032
2D T_1*ρ*_	1.1	0.55	0.35	0.21
2D T_2_	^a^	0.99	^a^	−0.025

The contrast differences in various imaging techniques (3D T_1_, 3D T_1*ρ*_, 2D T_1*ρ*_, and 2D T_2_) when comparing different ROIs by calculating RRTD values. The values highlight the sensitivity of each technique in differentiating between ROIs, with higher values indicating greater contrast and, consequently, better differentiation.

^a^Not investigated.

### Histopathology showed fibrosis and early fibrosis in the MI area

**Figure 8:**
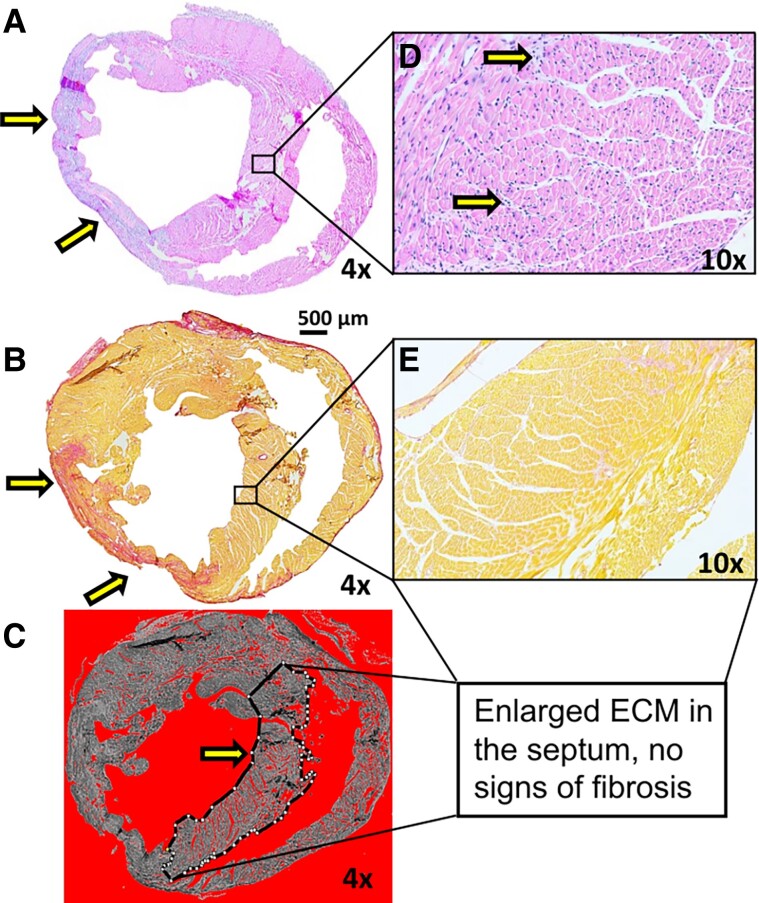
(*A*) HE-stained cross-section of myocardium showed loss of cardiomyocytes and coronary capillaries and abundant infiltration of inflammatory cells, mainly lymphocytes, macrophages, and neutrophils, 7 days after MI. (*B*) Additionally, the formation of scar tissue was detected by Picrosirius staining in the LV wall. (*C*) Increased interstitial oedema was detected globally in the myocardium, but especially in the septum area (dotted line). (*D*) HE-stained septum after MI. Interstitial oedema and necrotic cardiomyocytes in the septum after MI. No fibrosis (C and *E*) but increased extracellular matrix space was detected after MI in the septum. Corresponding magnifications are marked in the individual figures. Arrows are pointing to the damaged areas in the left ventricular wall and septum.

*Figure 8* shows the results of the histopathological staining from the same sample presented in *Figure 3*. The histopathological analysis showed loss of cardiomyocytes and capillaries with abundant infiltration of inflammatory cells (*Figure 8A*) and the formation of fibrosis (*Figure 8B*) in the anterior wall of the LV after 7 days of MI in comparison with healthy controls. In addition, increased interstitial oedema (*Figure 8C*) and increased necrotic cardiomyocytes were detected in the septum area after MI in the HE stained sections (*Figure 8D*). Because T_1*ρ*_ and T_2_ relaxation times were elevated in the septum, the septum area was carefully examined. Sirius Red (SR) staining did not show signs of elevated collagen content in the septum (*Figure 8E*); however, in two samples, early-phase fibrosis was detected in the septum.

## Discussion

In this study, a combination of 3D MB-SWIFT sequence and the CS image reconstruction method was used to image MnCl_2_ phantom and the myocardium after subacute MI in *ex vivo* mice hearts. The phantom study showed a linear relationship between relaxation rate and MnCl_2_ concentration for both T_1_- and T_1*ρ*_-weighted MB-SWIFT measurements.

The results indicated that the 3D accelerated relaxation time–weighted MB-SWIFT-CS is an accurate tool for imaging subacute MI in the myocardium quantitatively. The resulting image allows one to examine the heart from any direction with high spatial resolution. For example, the whole SAX direction is available with >100 views with a single view thickness of <0.1 mm, which is significantly thinner than the 1–2-mm slice thickness in most of the multi-slice pre-clinical studies and guidelines.^8,10,45,46^ Appropriately, the total scan time for endogenous relaxation time–weighted 3D measurement was <4 min.

The regularization parameters of the CS method were manually tuned, which is a subjective step. However, as overtly strong LLR regularization causes blurring and visible block structures, and too weak regularization quickly introduces noise in the images and quantitative maps (*Figures 3* and *4*) clear guidelines for the manual tuning step existed. Hence, we believe that the reconstructions are appropriately regularized.

In our study, there was no need to adjust regularization strength between 3D T_1_ and 3D T_1*ρ*_ reconstructions. The duration of the image reconstruction is entirely dependent on the computational efficiency of the hardware utilized. The reconstruction times here were somewhat lengthy but could be improved by running the reconstructions on, e.g. dedicated computational servers.

3D T_1_, 3D T_1*ρ*_, and 2D T_1*ρ*_ relaxation time constants were elevated in the LV wall, which is in line with the previous literature, showing that e.g. rotating frame relaxation time constants increase in the MI.^8,10,11^ T_1*ρ*_ relaxation time constants acquired by MB-SWIFT were slightly lower than T_1*ρ*_ relaxation time constants acquired with FSE. It has been shown earlier that T_1*ρ*_ values acquired by MB-SWIFT sequence tend to be lower even if the similar FSE acquisition is used.^20^ Interestingly, elevated T_1*ρ*_ relaxation time values were also observed in the septum, which may be caused by inflammation or early-phase of fibrosis.^47^ In the septum, HE showed macrophage infiltration, and optical analysis revealed oedema-related expansion of the extracellular space. The 2D T_2_ relaxation times were also elevated in the septum.^5,9,48^ However, in two samples, together with oedema and macrophage infiltration, the early phase of fibrosis (mild collagen formation) was detected in the septum area. For that reason, elevations of T_1*ρ*_ relaxation time values in the septum could be caused by a synergy of inflammatory factors, oedema, and collagen formation rather than inflammation alone. Overall, the histological analysis closely corroborated the CMR findings. However, with the current sample size, it is not possible to draw definitive conclusions about the generation of the T_1*ρ*_ signal. Both 2D CMR and pathohistological analysis support the conclusion that 3D T_1*ρ*_ is sensitive to inflammation and fibrosis, and it is possible to separate these regions based on the T_1*ρ*_ relaxation time maps.

CMR and histopathological findings suggest that 3D T_1*ρ*_ MB-SWIFT-CS has the potential to be utilized in the same areas as T_2_, as it allows for the assessment of inflammation and early-phase fibrosis in this mouse model. Because PFA affects the tissue properties of the myocardium, *in vivo* CMR and the comparison with the late gadolinium enhancement (LGE) method are unquestionably needed to truly evaluate the applicability of the method presented in this paper.

The RRTD analysis showed that 3D T_1*ρ*_ relaxation times were sensitive to different regions in the damaged myocardium. The T_1*ρ*_ relaxation times were remarkably elevated in the fibrotic areas compared with the remote ROIs, and a substantial difference in the T_1*ρ*_ relaxation time constants between the fibrotic and inflammation regions can also be found based on the RRTD values. Additionally, acquired 3D T_1*ρ*_ values in the remote and control myocardium are in the same range as reported earlier in *in vivo* studies with healthy human subjects.^13^ Additionally, RRTD analysis revealed a relevant difference in the 3D T_1_ relaxation times between the remote and control areas, which may be caused by underlying oedema. In 2D T_2_ measurements, RRTD indicated clear differences between tissue with oedema and remote tissue. However, RRTD between remote and control tissues was slightly negative, which may indicate that changes in remote tissue are likely driven by early inflammatory processes or collagen-related alterations, rather than variations in free water content. Our results suggest that T_1*ρ*_ could be more sensitive to subtle, early-stage alterations in remote tissue, which may be influenced by inflammation or early-stage fibrosis.

Since the minimum FOV size for the sequence was limited by our hardware, the small size of the mouse heart caused a great challenge in terms of resolution and overall image quality. However, despite the relatively large FOV, reasonable image quality could be achieved with the CS approach. Also, the number of samples was limited due to ethical considerations, which could affect the robustness of the statistical analysis.

Even though results for both 3D T_1_ and 3D T_1*ρ*_ measurements were satisfactory, imaging protocols still need further adjustments. For T_1_ measurements, the equilibrium net magnetization should be determined because it would allow improving the data processing for the map calculations, i.e. specifically the correction of the intermediate T_1eff_ would be more accurate. The utilized FA-based correction of T_1_ values is sensitive to the precision of the FA and thus sensitive to the accuracy of the B_1_ field. With higher FAs the T_1eff_ −> T_1_ correction becomes more sensitive and is less appropriate than a correction method utilizing the equilibrium magnetization.^32,34,49^ In this study, with FA of 4° we were able to maximize the SNR while still ensuring a stable and reliable T_1eff_ −> T_1_ correction. On the other hand, smaller FAs had to be used for the phantom measurements, since measurements with higher FAs yielded unrealistic T_1_ relaxation time constants for those samples by the unstable correction.

Statistically significant differences between fibrotic and remote tissue were detected based on the 3D T_1_ and 3D T_1*ρ*_ relaxation time maps. In addition to fibrosis, inflammation was also determined and separated from the remote myocardium based on the T_1*ρ*_ relaxation time maps. Our data also suggest that CS translates well to *ex vivo* 3D CMR, providing excellent groundwork for rapid and quantitative ultra-short TE *in vivo* applications in the future.

## Conclusion

The relaxation time–weighted MB-SWIFT-CS method overcomes many challenges associated with conventional 2D CMR by showing its ability to characterize smaller pathologies in the myocardium rapidly, quantitatively, and in 3D.

## Data Availability

The datasets used and analysed during this study are available from the corresponding author upon reasonable request.

## Lead author biography

Iida Räty is an early-stage researcher at the A.I. Virtanen Institute for Molecular Sciences at the University of Eastern Finland. Currently, she is working on her PhD thesis, focusing on developing quantitative cardiac magnetic resonance (CMR) methods. Her goal is to improve fast 3D cardiac magnetic resonance imaging by utilizing ultra-short echo time sequences, advanced image reconstruction techniques, and mapping methods (e.g., T1ρ relaxation time mapping and quantitative susceptibility mapping) in animal models and in high magnetic fields.

## Appendix 1

The T_1_-weighted image series was reconstructed with an LLR-regularized subspace constrained image reconstruction of the form:

Equation A.1

$$\hat{\alpha }=\underset{\alpha }{\arg\;\min}\;\|E\Phi_{K}\alpha -m{\|}_{2}^{2}+\lambda R(\alpha ),$$

Where α contains the subspace coefficient images, Φ*_K_* is the contrast dimension subspace basis with *K* basis vectors, *E* is the MRI forward operator, which maps the data from *k*-space to image space, *m* contains the binned *k*-spaces, *R* is the LLR regularization, and λ the parameter determining the strength of the regularization. After the image reconstruction, the original image series *u* can then be recovered as:

$$u\approx \Phi_{K}\alpha .$$

The subspace basis was determined by applying principal component analysis on a set of simulated signals. The signal relaxations for the T_1_-weighted acquisition can be written as follows:

Equation A.2

$$S(t)=A-A\;\exp\;\left(-\frac{t}{T_{1\mathrm{eff}}}\right).$$

A set of simulated signals was formed such that 1000 T_1eff_ values were linearly chosen from [500, 2700] and [500, 1300] ms for MnCl_2_ phantom and hearts, respectively. Additionally, for each T_1eff_ value, 100 values for *A* were also linearly chosen from [0.01, 1]. Principal component analysis was performed on the simulated signals and the first three principal components were chosen for the subspace basis.

T_1*ρ*_ images were reconstructed with

Equation A.3

$$\hat{u}=\underset{u}{\arg\;\min}\;\|Eu-m{\|}_{2}^{2}+\lambda R(u).$$

## Appendix 2

Three-dimensional T_1_ relaxation time maps were calculated as presented in the study by Zhang *et al.*.^21^ Effective T_1_ (T_1eff_) maps were generated pixel-by-pixel by utilizing three-parameter non-linear least squares fitting. The T_1eff_ can be determined as follows:

Equation B.1

$$S(t)=A-B\exp\;\left(\frac{t}{T_{1\mathrm{eff}}}\right),$$

where *S*(*t*) represent the signal, *A* and *B* are fitting parameters, and *t* is time. T_1_ maps can then be calculated as follows:

Equation B.2

$$T_{1}={\left(\frac{1}{T_{1\mathrm{eff}}}+\frac{\ln\;\left(\cos\;\varphi \right)}{\mathrm{TR}}\right)}^{-1},$$

Where *ϕ* and TR are FA and the pulse TR, respectively.

3D T_1*ρ*_ relaxation time mapping

In order to account for the effect of T_1_, the average steady-state signal intensity

Equation B.3

$$S(T_{\mathrm{mp}})=\frac{A_{\mathrm{ss}}(1-E_{1}^{m}\cos^{m}\varphi )}{2}\frac{1+e^{-\frac{T_{\mathrm{mp}}}{T_{1\rho }}}}{1-E_{1}^{m}\cos^{m}\varphi e^{-\frac{T_{\mathrm{mp}}}{T_{1\rho }}}},$$

was normalized and presented as follows:

Equation B.4

$$\tilde{S}(T_{\mathrm{mp}})=\frac{1+e^{-\frac{T_{\mathrm{mp}}}{T_{1\rho }}}}{1-E_{1}^{m}\cos^{m}\varphi e^{-\frac{T_{\mathrm{mp}}}{T_{1\rho }}}},$$

where *ϕ* is FA and $E_{1}=e^{\frac{-\mathrm{TR}}{T_{1}}}$. After the data was normalized, T_1*ρ*_ was acquired by minimizing

Equation B.5

$$\arg\;\;\min\|\tilde{Z}-\tilde{S}(T_{\mathrm{mp}}){\|}_{2}^{2},$$

where $\tilde{Z}$ is normalized measured signal.
